# SOC Strategies and Organizational Citizenship Behaviors toward the Benefits of Co-workers: A Multi-Source Study

**DOI:** 10.3389/fpsyg.2017.01740

**Published:** 2017-10-05

**Authors:** Andreas Müller, Matthias Weigl

**Affiliations:** ^1^Institute for Occupational, Social and Environmental Medicine, Medical Faculty, University of Düsseldorf, Düsseldorf, Germany; ^2^Work and Organizational Psychology, University of Duisburg-Essen, Duisburg, Germany; ^3^Ludwig-Maximilians-University, Munich, Germany

**Keywords:** performance, older workers, social support, SOC, successful aging, teamwork

## Abstract

**Background:** Individuals’ behavioral strategies like selection, optimization, and compensation (SOC) contribute to efficient use of available resources. In the work context, previous studies revealed positive associations between employees’ SOC use and favorable individual outcomes, like engagement and job performance. However, the social implications of self-directed behaviors like SOC that are favorable for the employee but may imply consequences for coworkers have not been investigated yet in an interpersonal work context.

**Objective:** This study aimed to assess associations between employees’ use of SOC behaviors at work and their organizational citizenship behaviors (OCB) toward the benefits of co-workers rated by their peers at work. We further sought to identify age-specific associations between SOC use and OCB.

**Design and Method:** A cross-sectional design combining multi-source data was applied in primary school teachers (age range: 23–58 years) who frequently teach in dyads. *N* = 114 dyads were finally included. Teachers reported on their SOC strategies at work. Their peer colleagues evaluated teachers’ OCB. Control variables were gender, workload, working hours, and perceived proximity of relationship between the dyads.

**Results:** We observed a positive effect of loss-based selection behaviors on peer-rated OCB. Moreover, there was a significant two-way interaction effect between the use of compensation strategies and age on OCB, such that there was a positive association for older employees and a negative association for younger employees. There were no significant main and age-related interaction effects of elective selection, optimization, and of overall SOC strategies on OCB.

**Conclusion:** Our study suggests that high use of loss-based selection and high use of compensation strategies in older employees is positively related with OCB as perceived by their colleagues. However, high use of compensation strategies in younger employees is perceived negatively related with OCB. Our findings contribute to a better understanding of the age-differentiated interpersonal effects of successful aging strategies in terms of SOC in organizations.

## Introduction

Due to the aging of the workforce in many countries, organizations seek ways to promote functioning and well-being of their employees throughout the whole work-life span. Research shows that individuals’ behavioral strategies of selection, optimization, and compensation (SOC, Baltes and Baltes, 1990) are key contributors, particularly in older individuals to cope effectively with dwindling individual resources (Riediger et al., 2006). Accordingly, previous studies in the work context revealed positive associations between employees’ SOC use and favorable individual outcomes, like engagement and job performance (for an overview, see Müller and Weigl, 2015; Moghimi et al., 2017). Moreover, first interventional approaches to train and apply SOC behaviors on the job were introduced, indicating that the SOC model is a promising approach for the development of occupational health and stress prevention measures drawing upon a life-span approach (Müller et al., 2016; Becker et al., 2017).

However, previous research exclusively considered individual effects of SOC, i.e., cognitive-behavioral, performance, or health outcomes. Implications on the inter-individual or organizational level are largely neglected (Moghimi et al., 2017). In modern work environments, collaboration and teamwork are essential. Both, individual gains and organizational benefits through application of individual behavioral strategies at work need to be in balance. This study therefore aimed to assess for the first time the associations between employees’ use of SOC behaviors at work and their organizational citizenship behavior (OCB) specifically toward the benefits of co-workers rated by their immediate peers at work.

We deem that this study contributes to the current knowledge base on effects of behavioral strategies of successful aging at work in three ways: first, since inter-individual effects of SOC are under-investigated, our study expands previous approaches on the use of SOC behaviors at work through its social perspective. To the best of our knowledge, no studies have yet surveyed the benefits or harms of individuals’ SOC use through the eyes of their immediate co-workers. In this vein, our findings help to understand the social implications of SOC. Second, our study seeks to further elucidate the differential and shared effects of the individual SOC strategies with age. Originally, it has been proposed that SOC use is most efficient in the concerted and concurrent application of all SOC strategies (Freund and Baltes, 2000). However, previous research suggested that single SOC strategies are more efficient in response to personal age-related changes and environmental demands (e.g., Demerouti et al., 2014; Riedel et al., 2015). Lastly, the majority of available research on the effects of SOC use relied on self-reports that are prone to bias (Moghimi et al., 2017). We therefore aimed to test the single as well as overall effects of SOC using different sources of data, i.e., self and peer ratings.

### SOC Behaviors

In the field of age and occupational functioning, concepts that consider aging in terms of a dynamic development of gains, losses, and the reorganization of resources serve as a base for explaining successful aging at work (Riediger et al., 2006; Zacher, 2015). One of the key concepts in this area is the model of SOC. It suggests that individuals aim to maintain an optimal allocation of individual resources, functioning in the face of challenges, and adaptation to declined resources (Baltes and Baltes, 1990; Riediger et al., 2006).

The core propositions of the SOC model state that individuals manage age-related changes and losses of capabilities and resources more efficiently by virtue of three interrelated action strategies (Freund and Baltes, 2002b): *Selection* behaviors aim to focus resources on specific goals in contrast to allocating resources among multiple goals. Thus, selection determines the direction of personal development and resource investment. Selection can be differentiated into either elective, that is, directed toward desired future states (e.g., an employee decides to exclusively pursue one important goal at work, e.g., attaining a specific position within the organization) or loss-based, that is, directed at the reorganization of goals in response to perceived problems or experienced challenges (e.g., an employee decides to change job tasks because the current job demands can no longer be accomplished). *Optimization* behaviors aim to facilitate individuals in obtaining and continuously improving the means to successfully pursue a desired goal (e.g., an employee acquires the necessary competencies to successfully perform important job tasks). Thus, optimization refers to the quality as well as to the persistence of resource allocation in service of goal pursuit. As third SOC behavior, *compensation* includes the acquisition and application of alternative means to achieve a desired goal in the case of obstacles or resource losses (e.g., an employee with chronic diseases seeks opportunities of extra support and additional assistance at work). As such, compensation specifically refers to the flexibility of resource allocation in the pursuit of goals. In summary, the SOC model proposes that the use of SOC behaviors is particularly effective when individuals focus on fewer, but more important goals, pursue these goals in an optimized manner, and, in doing so flexibly apply adequate compensatory means to address goal-relevant barriers (Baltes, 1997).

Main aspects of the SOC model are in accordance with the tenets of motivational theory on life-span development (Heckhausen et al., 2010): both approaches assume that the agency of individuals is the driver of human development and functional adaptation. Moreover, the SOC model and the motivational theory on life-span development agree that adaptive life course development involves the selection and disengagement from goals.

### SOC and Successful Aging at Work

During the past decade, SOC-based research has been established as a powerful approach for explaining organizational behavior related to coping with age-related changes in individual resources across the work lifespan (cf., Müller and Weigl, 2015). Previously, Moghimi et al. (2017) synthesized all available research on SOC use at work. They concluded that SOC behaviors are important for various employee outcomes, particularly for job performance, job satisfaction, and engagement. However, in their comprehensive review, Moghimi et al. (2017) also identify remaining gaps in the current evidence base on SOC use in the workplace. One particular shortcoming refers to contextual outcomes of SOC on the organizational level. For example, it has been suggested, that employees’ SOC use has consequences for teams, and organizations (Baltes and Dickson, 2001). However, the empirical investigation of this assumption has not been undertaken yet and deserves in-depth exploration in work settings (Moghimi et al., 2017).

### Organizational Citizenship Behaviors

A second focal construct in our study is employees’ OCB. It can be defined as discretionary employee behaviors or extra-role behaviors that support coworkers, contribute to team functioning and to the organization (Borman and Motowidlo, 1997). OCB has been considered as employee activities that support the social and organizational environment beyond the actual core task or job role (Podsakoff et al., 2009). Hence, OCB includes extra-role activities or behaviors that are not just about carrying out one’s prescribed job requirements, i.e., in-role job performance. The construct of OCB has received broad attention throughout the past decade and it has been shown that it is a key variable in employees’ organizational behavior and a meaningful measure of organizational effectiveness. The literature on OCB and potential consequences showed that employees’ OCB may be associated with important individual- and organizational-level outcomes (Podsakoff et al., 2009).

Although several conceptualizations of OCB have been introduced, Williams and Anderson’s (1991) distinction of extra-role behaviors into two major categories is one of the most accepted and established (Podsakoff et al., 2009). Williams and Anderson (1991) differentiated between OCBs directed toward the benefits of the employing organization (e.g., taking extra shifts) and OCBs directed toward the benefits of other individuals (e.g., supporting colleagues). The latter dimension captures behaviors of interpersonal helping or facilitation. In our study, we focused on this interpersonal dimension of OCB, specifically the OCB toward the benefits of co-workers and the team.

### The Association between SOC and OCB Directed toward the Benefits of Co-workers

Meta-analytic evidence shows that SOC is positively correlated with both self-reported and externally rated job performance (Moghimi et al., 2017). However, available studies mainly focused on indicators of in-role job performance, like productivity and efficiency (Abraham and Hansson, 1995; Yeung and Fung, 2009), performance quality in nursing (Baethge et al., 2016), supervisor rated overall job performance (Bajor and Baltes, 2003), or fulfillment of formal requirements of the job (Demerouti et al., 2014). To the best of our knowledge, empirical investigations on the associations between SOC and extra-role job performance in terms of OCBs that are directed toward the benefits of other co-workers are missing.

Previous research indicates that exchange relationships play an important role as OCB antecedents (Cardona et al., 2004). From the perspective of social exchange theory, persons follow certain explicit or implicit rules of exchange, which evolve over time into perceptions of trust, justice, and mutual commitment (e.g., Cropanzano and Mitchell, 2005). One of the most important universal rules of social exchange is reciprocity, which means that people should return favors, e.g., support or contributions that they received from others (Eisenberger et al., 2001). We assume that the use of SOC has an impact on the social exchange between co-workers and is consequently associated with OCB. For example, it can be assumed that the selection of a specific work task by a team member will have an impact on the perception of reciprocity within the team. Specifically, selection can be evaluated positively and supportive through the eyes of a team member, when an employee selects and carries out an unpleasant task. In contrast, selection might be perceived negatively in case selection means to disregard such a task, which has then to be taken over by a team-member.

Hypotheses about the direction of the association between SOC and OCB can be drawn from the perspective of resource allocation and conservation of resources (e.g., Hobfoll, 2002). From this perspective, efficient use of resources through SOC saves or establishes “spare” resources to apply extra-role behaviors like OCB toward the benefits of co-workers. For example, SOC at work involves behaviors like setting priorities to carry out the most important tasks first (selection), or informing oneself about the current state of the professional knowledge (optimization; see Müller et al., 2013). The application of these behaviors enables employees to efficiently achieve core task objectives. Through saving efforts in goal-directed behaviors, saved individual resources can be used to help co-workers. As example, if a teacher focuses on specific subjects, this may enable her/him to save time and efforts in preparation and teaching, what eventually allows her/him to invest additional time in communication and exchange with pupils, parents, and colleagues. This can eventually help to foster a collaborative and supportive learning climate which is beneficial for the team.

From the perspective of motivational theory on life-span development (Heckhausen et al., 2010), the association between SOC behaviors and OCB may differ depending on whether the selected goal is to maintain a good relationship to co-workers or to promote one’s personal career. Although the SOC model is unspecific about goal contents, previous findings provide first support that the use of SOC behaviors at work is positively related with socially desirable behaviors. For example, Freund and Baltes (2002b) reported that the use of SOC behaviors is positively related with important social aspects of successful life management like establishing a positive relationship with others. Moreover, the same study reported that the use of SOC behaviors also correlated with personal characteristics like conscientiousness, emotional stability, and openness. There is meta-analytic evidence showing that these personal characteristics in turn are important preconditions for OCB toward the benefits of co-workers (Chiaburu et al., 2011). We therefore assume that the use of SOC at work is positively related with OCB toward the benefits of co-workers.

*Hypothesis 1*: The use of SOC at work is positively related with OCB toward the benefits of co-workers.

### Age Effects on the Association between SOC and OCB

We furthermore assume that the above hypothesized relationship between SOC use and OCB is moderated through employees’ age. Drawing on socioemotional selectivity theory (SST, Carstensen et al., 1999) we suggest that older employees’ SOC use might be more beneficial in establishing effective social relationships at work, what, eventually, results in increased OCB-related outcomes. SST postulates that the individual perception of future time perspectives (i.e., the expectation of how much time is left in our life) is related to the choice of goals (Carstensen et al., 1999). This includes particularly a reorganization of motivational focus toward establishing emotionally satisfying social relations in older persons, whereas in younger persons the instrumental function of social relations, for example, in terms of promoting future opportunities, plays a greater role (Luong et al., 2011). Accordingly, Freund and Blanchard-Fields (2014) observed consistently across four studies, that older persons exhibited stronger altruistic values and exhibited more helping behaviors compared to younger persons. Moreover, research on age and social experience indicates that older persons often attained a greater sensitivity to interpret the needs of other persons and to foresee the social implications of their own behaviors (Hess, 2006).

With regard to work environments, previous research suggested that the employees’ age is negatively related to their occupational future time perspective, i.e., employees’ perceptions of their remaining time at work and their career opportunities (for an overview, see Henry et al., 2017). Moreover, a study of Treadway et al. (2010) observed that socially competent employees with more shallow occupational future time perspectives engage in more altruistic and other-centered networking behaviors than employees with longer occupational future time perspective. In line with this research, we assume that older employees *are more motivated* to select SOC behaviors at work that are geared toward benefits of their co-workers. Moreover, due to accumulated social experience, older employees might *be better able* to choose the best means for attaining goals in a socially acceptable way (see also Sonnentag, 2000; Kanfer and Ackerman, 2004). In the case of school teachers, experienced teachers may have a larger skill set to master difficult and challenging relationships with pupils what eventually helps to maintain professional and supportive relationships in the school. We therefore hypothesize that the positive association between the use of SOC and OCB toward the benefits of co-workers is stronger with higher age.

*Hypothesis 2*: The positive association between SOC at work and OCB toward the benefits of co-workers is moderated by age, such that this relationship is stronger for older employees.

### Exploration of Differential Effects of Individual SOC Strategies on OCB

Originally, it has been proposed that SOC is most effective when individuals use all SOC strategies in a joint and coordinated way (e.g., Freund and Baltes, 2000). However, previous research indicates that the SOC strategies are also representing distinct action processes with independent and differentiated effects on outcomes such as performance and work ability (e.g., Abraham and Hansson, 1995; Freund and Baltes, 2002b; Wiese et al., 2002; Bajor and Baltes, 2003; Yeung and Fung, 2009; Demerouti et al., 2014; Riedel et al., 2015; Zacher et al., 2015). From the perspective of action theory, both selection strategies are related to the choice of goals, whereas optimization and compensation are directed to establish the adequate means to achieve goals (Freund and Baltes, 2000). Taking the perspective of cognitive theories of control (e.g., Hockey, 1997), Demerouti et al. (2014) it can be assumed that the use of selection strategies has a negative impact on extra-role behaviors in terms of adaptivity to change because employees strive to execute high priority in-role tasks first, particularly when resources are low. Demerouti et al. (2014) further suggest in accordance with conservation of resources theory (Hobfoll, 2002) that optimization and compensation are positively related to in-role performance as well as to extra-role performance. Since both refer to means to attain task goals, they potentially enhance the use of available resources (Demerouti et al., 2014).

Moreover, previous literature emphasized that loss-based selection and compensation are both “responses” to external or internal circumstances (e.g., hindrances) which require flexible goal adjustment or adaptive adjustment of means (e.g., Freund and Baltes, 1998, 2002a). From the viewpoint of research on social support, particularly this responsive nature of both strategies needs to be considered (Maisel and Gable, 2009). Findings suggest that responsiveness, in terms of adapting one’s own behaviors to meet the needs of a partner, is an important indicator for perceived social support (e.g., Neff and Karney, 2005; Maisel and Gable, 2009). Translating these findings to the social context of SOC at work, it can be argued that particularly employees with high use of loss-based selection or compensation are more responsive in their behaviors, i.e., being capable to flexibly adjust to their co-workers’ needs. With regard to our study questions, this line of research suggests that loss-based selection and compensation might be positively associated with to OCB toward the benefits of co-workers.

### Exploration of Age Effects on the Association between Individual SOC Strategies and OCB

Concerning the role of age, the following considerations led us to explore the social effects of the four individual SOC strategies and among different age groups. Previous research shows that not all SOC strategies are equally beneficial at all stages of adulthood (e.g., Riediger et al., 2006). Due to losses and shrinking time perspectives, the number of unattainable personal goals potentially increases in older age (e.g., Carstensen et al., 1999). In the same vein, in older employees the likelihood increases that important means for goal achievement are no longer available. From the perspective of conservation of resources (Hobfoll, 2002), the use of the two loss-related SOC strategies, loss-based selection and compensation, should be more beneficial for older employees than for their younger colleagues to allocate remaining resources in an adaptive way. Accordingly, a study of Wrosch et al. (2003) showed that, disengagement from unattainable goals and reengagement in new and meaningful goals is associated with high well-being, particularly in older individuals. With respect to our study, we therefore assume that the use of loss-based selection and compensation helps particularly older employees to allocate their resources more efficiently in order to exert extra-role behaviors like OCB toward the benefits of co-workers.

Overall, the knowledge base on the sequelae of the individual SOC strategies on OCB is inconsistent and remains inconclusive. We therefore refrained from formulating specific hypotheses about the effect of individual SOC strategies. This exploratory approach generates preliminary empirical findings that provide the base for future studies on social effects of SOC strategy use.

## Materials and Methods

### Study Design, Setting, and Participants

The data was derived from a cross-sectional study combining multi-source data from primary school teachers, who frequently teach in dyads, i.e., class approach of team teaching. Hereby, two teachers concurrently and collaboratively teach the class. Additionally on a weekly level, teachers are requested to commonly plan and prepare teaching with their peer teacher, discuss didactic approaches with their peer, as well as jointly reflect problems or potential challenges with pupils or parents. The data was collected between May and June 2012 in eight primary schools in South Tyrol (Italy). The data collection was approved by the directorates of each school and all participants were informed about aims and procedures of the study.

### Procedure

Each teacher of the participating schools received an envelope with a main (for her-/himself) and a second, short questionnaire (for their team teaching colleague). Overall, 180 pairs of questionnaires were distributed. All teachers were asked to fill out their main, first questionnaire to report the use of SOC behaviors and to provide further sociodemographic characteristics. Furthermore, all teachers were asked to hand the second, separate questionnaire on to their team colleague. In the instruction, it was stated that they hand out the second questionnaire to their immediate colleague with whom they frequently perform team teaching. Within the instruction of the second survey sheet, the team colleague was asked to fill in the short questionnaire to evaluate the first teacher’s OCB. Both teachers were required to fill in and return their questionnaires independently. To maintain confidentiality, with each questionnaire a sealed return envelope was attached. Each pair of questionnaires was prior marked with an identical code to ensure the matching of the pairs.

### Measures

#### SOC Behaviors

To measure the use of SOC strategies, a short German version of the original SOC questionnaire (Baltes et al., 1999; Freund and Baltes, 2002b) was used. In the present study, we used the revised response scale developed by Zacher and Frese (2011). It comprises the four sub-scales that respectively characterize specific SOC behaviors: elective selection, loss-based selection, optimization, and compensation. Each sub-scale consists of three items, which were rated on a 5-point Likert scale from 1 = “does not apply at all” to 5 = “applies completely.” A sample item for selective election behaviors is “I concentrate all my energy at work on few things”; for loss-based selection “If I can’t do something important at work the way I did before, I look for a new goal”; for optimization behaviors “If something at work matters to me, I devote myself fully and completely to it”; and for compensation “When things at work don’t go as well as they used to, I keep trying other ways until I can achieve the same result I used to.” We assessed the reliability with McDonald’s (1999) Omega using a bias-corrected and accelerated bootstrap approach (Kelley and Cheng, 2012). Omega is based upon the sum of squared loadings on one common factor. We used McDonald’s coefficient Omega because it is compared to Cronbach’s alpha less biased when assumptions of essentially tau equivalency are not met, which is usually the case for most measures (Dunn et al., 2014). McDonald’s Omega for the total SOC scale is 0.77, 95% CI 0.69–0.82. For the individual scales: elective selection = 0.65, 95% CI 0.48–0.76, loss-based selection = 0.73, 95% CI 0.60–0.81, optimization = 0.52, 95% CI 0.34–0.63, and compensation = 0.63, 95% CI 0.45–0.73.

#### Organizational Citizenship Behaviors (Peer Rating)

To evaluate first the teacher’s extra role and team behaviors, a short version of the validated role-based performance scale of Welbourne et al. (1998) was applied. We used an adapted German 4-item version that refers to employees’ contributions to team functioning. Example items are: “Doing things that help others when it’s not part of his/her job,” “Helping so that the team is a good place to be,” and “Making sure his/her work group succeeds.” All items were answered on a 5-point Likert scale from 1 = “Needs much improvement” to 5 = “Excellent.” Cronbach’s alpha for the total OCB scale was excellent with α = 0.92.

#### Age

Information on teachers’ age was based on a single question in the survey: “How old are you (in years)?” Answers were provided in free text.

#### Control Variables

To control for potential influences of individual and task-related characteristics, several control variables were included in the questionnaire. All teachers were asked for gender (1 = female, 2 = male), their working hours (in hours per week), and job tenure (in years; “How long are you working in your current profession?”). Additionally, all teachers were asked to rate their job demands since high or overtaxing requirements on the job may force employees to develop and apply respective behavioral strategies on the job to cope with excessive loads or time pressure. We used an abbreviated two-item scale of a validated scale from a German instrument on Work Analysis that assesses work overload (TAA) (Glaser et al., 2015). An example is “Frequently, there is too much work at once.” Internal consistency was good (α = 0.70). To assess the perceived proximity of the relationship between the teacher and his/her peer, the second teacher was asked the following question: “How well do you know the colleague?” We included this measure since we assumed that in close or familiar working dyads, potential bias for overly positive OCB ratings may occur. Responses were provided on a 5-point Likert scale from 1 = “not well at all” to 5 = “very well.”

### Statistical Analyses

In the first step, descriptive and inferential statistical analyses were conducted. Hypotheses were tested with hierarchical moderated multiple regression analysis (Cohen et al., 2003). SOC, age, and OCB were used as continuous variables in the analyses. In step 1, we included the control variables gender, workload, working hours, and perceived familiarity with the colleague. In step 2, we determined the main effects of SOC and of chronological age on peer-rated OCB. In step 3, we additionally included the interaction terms of SOC use and chronological age. Finally, this procedure was repeated with the four individual SOC strategies. As recommended, all continuous variables were mean-centered before calculating interaction terms. Tests and graphical displays for slope differences used group differences with ±1 SD from the mean. Statistical significance was set at *p* < 0.05. All analyses were performed using SPSS 24.0 (IBM Inc., Chicago).

## Results

One hundred and fifteen questionnaires were returned to the study team. The majority of the sample were female (*n* = 109, 94.8%) what fairly represents the overall gender distribution among primary school teachers in South Tyrol. Additionally, 120 peer ratings were sent back. Finally, *N* = 114 dyads, i.e., matched pairs of questionnaires and peer ratings, were included into the further analyses. This equates to a response rate of 63.3%.

### Descriptive Statistics

**Table 1** reports the descriptive results for sample characteristics and study variables. Teachers’ mean age was *M* = 41.75 (SD = 9.39) with a range between 23 and 58 years. 13.3% of the teachers were below 30 years old (*n* = 15) and 15.9% (*n* = 26) were older than 50 years. Mean job tenure was *M* = 20.58 years (SD = 10.80) with a range between 1 and 39 years. Since age and job tenure were highly correlated (*r* = 0.90, *p* < 0.001), we excluded job tenure from further analyses.

**Table 1:** Means (*M*), standard deviations (*SD*), and intercorrelations of study variables.

	*M*	*SD*	1	2	3	4	5	6	7	8	9	10
1 OCB (peer rating)	4.16	0.77										
2 Gender	1.05	0.22	0.00									
3 Weekly working hours	32.52	8.71	0.05	-0.34**								
4 Proximity of relationship (peer rating)	4.13	0.66	0.25**	-0.11	0.02							
5 Job demands	3.34	0.81	0.03	0.07	0.21*	0.03						
6 Age	41.75	9.39	0.04	-0.07	0.21*	0.31**	0.06					
7 SOC (overall scale)	3.71	0.46	0.14	0.02	-0.09	0.18	-0.04	0.22*				
8 SOC: elective selection	3.53	0.70	-0.04	0.03	-0.12	0.14	-0.01	0.23*	0.54**			
9 SOC: loss-based selection	3.68	0.79	0.20*	-0.04	-0.07	0.11	-0.18	0.23*	0.80**	0.29**		
10 SOC: optimization	3.78	0.57	0.08	0.09	-0.01	0.09	0.15	0.10	0.65**	0.17	0.33^∗∗^	
11 SOC: compensation	3.85	0.69	0.15	0.00	-0.06	0.22*	0.02	0.14	0.71**	0.05	0.46^∗∗^	0.36^∗∗^


***N* = 114. For gender, 1 = female, 2 = male. SOC, selection, optimization, and compensation. ^∗∗^*p* ≤ 0.01; ^∗^*p* ≤ 0.05, two-tailed.**

Next, intercorrelations of the sociodemographic and study variables were computed (cf., **Table 1**). With increased familiarity or proximity of the team teaching relationship, peers rated OCB behaviors more favorably (*r* = 0.25, *p* = 0.008); but there was no significant association with the use of overall SOC behaviors at work (*r* = 0.18, *p* = 0.061). Reported compensation behaviors were positively related to perceived proximity (*r* = 0.22, *p* = 0.021). Job demands were positively correlated with weekly working hours (*r* = 0.21, *p* = 0.039) but neither with the use of overall SOC behaviors at work nor with any of the individual SOC strategies; although the strength of the relationship between loss based selection and job demands was close to our significance criterion (*r* = -0.18, *p* = 0.055).

### Hypotheses Testing

For testing our two proposed hypotheses, we conducted regression analyses and controlled for gender, weekly work time, proximity of the relationship, and job demands. Our first hypothesis assumed a main effect of the use of SOC behaviors at work on OCB such that increased SOC behaviors are associated with increased peer-rated OCB (hypothesis 1). We tested for overall SOC behaviors as well as for each individual SOC strategy respectively. We obtained no significant association between the use of overall SOC behaviors at work and peer-rated OCB (β = 0.13, *p* = 0.227). Concerning the relationships between individual SOC strategies and OCB, we observed one significant association: increased loss-based selection behaviors were positively related to peer-rated OCB (β = 0.24, *p* = 0.021). The three other dimensions of SOC were not significantly related to OCB (elective selection: β = -0.07, *p* = 0.532; optimization: β = 0.03, *p* = 0.760; compensation: β = 0.11, *p* = 0.310). The significant effect of loss-based selected remained significant after removal of all control variables (β = 0.20, *p* = 0.040). The non-significant associations for the other three SOC strategies remained unaffected as well without adjusting for controls.

For the next step, we tested if teachers’ age had direct effects on SOC behaviors and peer-rated OCB. After controlling for the above listed confounders, age was not associated with OCB (β = -0.06, *p* = 0.569). However, age was significantly associated with overall SOC behaviors such that teachers with higher age reported more overall SOC behaviors (β = 0.29, *p* = 0.007). With regard to individual SOC strategies, age was significantly related to selection behaviors (elective selection: β = 0.30, *p* = 0.007; loss-based selection: β = 0.23, *p* = 0.040) but not to the two other strategies (optimization: β = 0.20, *p* = 0.069; compensation: β = 0.10, *p* = 0.345).

Our second hypothesis proposed a moderating influence of age for the relationship between SOC and peer-rated OCB (hypothesis 2). As depicted in **Table 2**, the association of overall SOC and OCB was not affected by teachers’ age (β_age × SOC_ = 0.15, *p* = 0.147).

**Table 2:** Effects of overall SOC and age on peer-rated organizational citizenship behavior (OCB).

		Outcome: OCB			
		
		*B*	95% CI *B*	β	*P*
Control variables	Gender	0.38	-0.40 to 1.16	0.11	0.330
	Weekly working hours	0.07	-0.12 to 0.25	0.09	0.461
	Familiarity with colleague	0.16	-0.03 to 0.35	0.18	0.103
	Job demands	0.01	-0.15 to 0.18	0.02	0.868
Age	Age	-0.08	-0.26 to 0.11	0.16	0.420
SOC use	SOC (overall)	0.13	-0.04 to 0.31	-0.09	0.139
Age × SOC	Age × SOC (overall)	0.12	-0.04 to 0.29	0.15	0.147


***N* = 114. All continuous variables were centered at their means. SOC, selection, optimization, and compensation. *B*, unstandardized regression coefficient; β, standardized regression coefficient; displayed are all results of step 3 (Model 3).**

**FIGURE 1:**
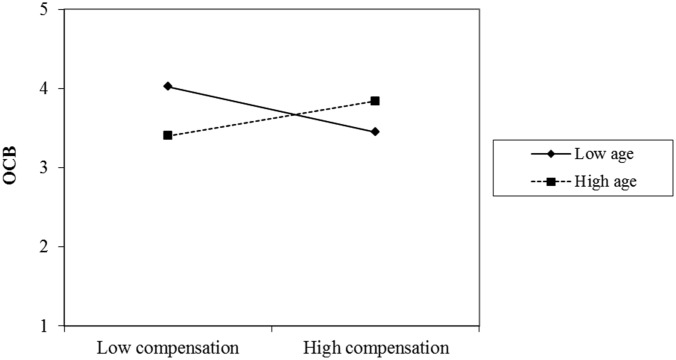
Moderating effects of chronological age on the relationship between the use of compensation strategies and peer-rated organizational citizenship behavior (OCB).

However, there was a significant two-way interaction effect between the use of compensation strategies and age on OCB (β_age × SOC compensation_ = 0.25, *p* = 0.043). This interaction effect is displayed in **Figure 1**. It shows that the positive association between SOC compensation and OCB was affected by teachers’ age: in older teachers, this relationship was positive (β = 0.18, *t* = 2.81, *p* = 0.006), whereas in younger teachers the association was negative (β = -0.23, *t* = -3.69, *p* < 0.001).

There was no age-related moderation effect between the other SOC strategies and peer-rated OCB (β_age × SOC elective selection_ = 0.15, *p* = 0.301; β_age × SOC loss based selection_ = -0.01, *p* = 0.950; β_age × SOC optimization_ = -0.16, *p* = 0.276). All results of the final step of the regression analyses for the individual and shared effects of the SOC strategies, age, and OCB are depicted in **Table 3**.

**Table 3:** Effects of individual SOC strategies and age on peer-rated organizational citizenship behavior (OCB).

		Outcome: OCB			
		
		*B*	95% CI *B*	β	*p*
Control variables	Gender	0.44	-0.35 to 1.22	0.12	0.269
	Weekly working hours	0.04	-0.15 to 0.23	0.05	0.663
	Familiarity with colleague	0.12	-0.08 to 0.32	0.14	0.232
	Job demands	0.08	-0.09 to 0.25	0.10	0.345
Age	Age	-0.05	-0.24 to 0.15	-0.06	0.632
SOC strategies	SOC: elective selection	-0.16	-0.37 to 0.05	-0.19	0.134
	SOC: loss-based selection	0.27	0.05 to 0.49	0.33	0.017
	SOC: optimization	-0.06	-0.27 to 0.15	-0.07	0.571
	SOC: compensation	-0.03	-0.23 to 0.18	-0.03	0.796
Age × SOC strategies	Age × elective selection	0.12	-0.11 to 0.35	0.15	0.301
	Age × loss-based selection	-0.01	-0.24 to 0.23	-0.01	0.950
	Age × optimization	-0.11	-0.31 to 0.09	-0.16	0.276
	Age × compensation	0.21	0.01 to 0.40	0.25	0.043


***N* = 114. All continuous variables were centered at their means. SOC, selection, optimization, and compensation. *B*, unstandardized regression coefficient; β, standardized regression coefficient; displayed are all results of step 3 (Model 3).**

To test the robustness of this observation we repeated this analysis and tested if the observed interaction terms remained significant after removal of the control variables. The crude estimate of the interaction effect of age and SOC compensation behaviors for OCB remained significant (β_age × SOC compensation_ = 0.21, *p* = 0.026).

## Discussion

Drawing on a sample of dyads of school teachers, this study sought to determine the individual and shared associations between behavioral strategies of successful aging at work in terms of SOC and OCB toward the benefits of co-workers. Our results show that the use of overall SOC behaviors was unrelated to OCB. However, the use of loss-based selection behavior is positively related with co-workers evaluations of OCB. Moreover, we observed that age moderates the association between the use of compensation strategies and OCB, such that there is a positive association between SOC compensation and OCB for older employees and a negative association for younger employees. No moderating effects of age were observed in respect to overall SOC behaviors and the remaining SOC sub-strategies. Although we found no confirmation for our two hypotheses and only two out of ten potential associations between overall SOC and OCB were statistically significant, our study contributes to the current knowledge base on SOC behaviors at work in various ways:

First, our study provides empirical evidence that the use of single SOC behaviors at work, specifically, loss-based selection and compensation behaviors in older employees, are positively associated with desirable social behaviors at the work place. So far, research neglected potential social consequences of SOC at work (Moghimi et al., 2017). Since self-directed SOC behaviors may impose a burden on co-workers, e.g., by neglecting assigned tasks that need to be completed, this might negatively affect perceived OCB that is directed toward the benefits of co-workers. This assumption has been empirically confirmed in recent research on the negative relationship between individual work behaviors and colleagues well-being (Tims et al., 2015). With the exception of compensation in younger employees, our findings provide no indication that individualized work behaviors in terms of SOC have an adverse effect on the social level or social relations at work.

Second, our study further elucidates the shared and differential influences of employee age for social consequences of employees’ SOC behaviors. Our results suggest that only responsive, loss-related SOC strategies, i.e., loss-based selection as well as compensation behaviors, have potentially positive effects on OCB toward the benefits of co-workers. The surveyed elective and growth-related strategies, i.e., elective selection and optimization, represent more persistent behaviors and were shown not to be associated with peer-rated extra-role behaviors. So far, loss-based selection and compensation have been mainly interpreted as responses to perceived decline of resources that force the individual either to adjust its goal system or to develop alternative means to maintain a desired level of functioning (Freund and Baltes, 2000). Our findings complement this perspective by suggesting the additional interpretation, that the use of both action strategies can also be interpreted as a response to social requirements or role expectations (see Neff and Karney, 2005; Maisel and Gable, 2009). Thus, employees with high use of loss-based selection might be better able or more willing to detach from personal goals when it is demanded from peers or the social context. Taken together, this hypothesis generated from our findings is worthwhile to be investigated in future studies.

Third, our findings contribute to a better understanding of the psychological processes of extra role performance in aging employees. Meta-analytic evidence revealed a positive association between age and OCB (Ng and Feldman, 2008). Drawing on the propositions of SST (Carstensen et al., 1999) and research on age and social experience (Hess, 2006), our results suggest that older employees with high use of compensation strategies might be more motivated or able to flexibly adapt their actions to the needs of others. In contrast, high use of compensation strategies in younger employees was associated with lower OCB toward the benefits of co-workers. *Post hoc*, we assume that younger teachers were either less willing or able to adjust their actions to social requirements or role expectations. Alternatively, compensation behaviors among younger teachers were yet not well adapted or implemented to their collaborative work routines what eventually resulted in inferior peer ratings. No age-related effects were observed for overall SOC behaviors or any of the over individual SOC strategies. Further research is therefore necessary to elucidate the determinant role of age in the interplay of behavioral strategies of aging in workplace and activities toward the benefits of co-workers and the team.

Fourth, our inclusion of peer ratings further contributes to more methodologically rigorous research in the field of SOC behaviors at work. So far, only a few investigations used multiple data sources to avoid common source bias: Bajor and Baltes (2003) showed that the use of SOC behaviors at work correlated with supervisor-rated job performance. Weigl et al. (2013) found a three-way interaction effect of age, job control, and use of SOC behaviors on supervisor-rated work ability of nurses. Yeung and Fung (2009) reported that the use of the SOC behaviors at work in older employees was positively associated with sales increases when tasks were not difficult or moderately difficult. Our findings provide additional evidence that the positive effects of SOC behaviors at work cannot be attributed to single source bias and spurious estimates. Moreover, and to the best of our knowledge, our approach to draw upon dyadic working relationships is the first study that directly assesses the social consequences of SOC behaviors through the eyes of co-workers.

### Limitations

First, our findings cannot be generalized to other professions without further consideration, because we focused on a convenience sample of teachers of different primary schools. The specific job conditions of our surveyed sample and the extent of team work and collaboration as well as task interdependencies may limit the external validity of our results. Future studies are necessary to replicate our findings in the same as well as in other professions and work contexts.

Second, we cannot draw conclusions about the effects of motivational states among older and younger teachers. The SOC model and its measures are unspecific about various aspects of motivation like goal content, and congruence of goals, because it assumes that goal selection and compensation are per se adaptive. SOC theory has been criticized for that (Heckhausen et al., 2010). Future research on SOC in occupational settings might therefore incorporate information on goal content and adequacy of goals (e.g., Stamov-Roßnagel and Biermann, 2012).

Third, the cross-sectional design does not allow inferences about underlying causal effects. For example, compensation strategies also include seeking social support from co-workers. Thus, from the perspective of social-exchange theory it might be that OCB toward the benefits of co-workers is a precondition to apply compensation behaviors. Moreover, the validity of age effects may be limited due to differences between birth cohorts (Smola and Sutton, 2002). Consequently, future studies should apply longitudinal or cohort-sequential designs.

Fourth, peer ratings may be prone to selection bias as well as to the likelihood to avoid negative ratings. Moreover, we did not attempt to establish a full data structure such that both teachers identically provide full information concerning their SOC use, OCB, and age. We thus cannot exclude bias arising from instances where the same teachers provided SOC information as well as evaluations concerning the OCB of their team teaching partner. Future investigations should therefore seek to establish complete dyadic data structures that allow for analyses of actor-partner interdependence models (Kenny et al., 2006).

Fifth, we acknowledge that our measure of extra role behaviors is not identical with conventional measures of OCB. We, however, assumed that in the context of teachers, our measure may reflect well the team-related behaviors and beneficial actions toward the newly introduced teaching teams in this occupational context. We strongly recommend that future studies incorporate measures that assess the key characteristics of OCB (Podsakoff et al., 2009).

Sixth, the rather low internal consistencies of the SOC subscales (and particularly that for optimization behaviors) limit the statistical power of our study. This suboptimal reliability of the SOC scale is consistent with previous studies (e.g., Wiese et al., 2000; Riedel et al., 2015). We tested the deletion of one problematic item of the optimization subscale (i.e., second question) but achieved no substantial improvement (i.e., McDonald’s Omega = 0.63, 95% CI 0.48–0.74). Notwithstanding, we reran all hypotheses tests with a revised, two-item optimization scale. Above-reported results did not change meaningfully; the association between the revised optimization scale and OCB remained insignificant (β = -0.06, *p* = 0.542), whereas age was significantly related to optimization behaviors (β = 0.29, *p* = 0.009). The interaction term of age and the revised optimization measure was insignificant again (β = -0.14, *p* = 0.340). The significant interaction of age and compensation was confirmed (β = 0.25, *p* = 0.047). Finally and for the sake of comparability between studies, we decided to report all results with the original 3-item optimization measure, but acknowledge that future investigations should aim to apply improved and reliable measures for all SOC components.

Finally, previous research discussed the dark side of OCB behaviors (for a review, see Bolino et al., 2013). For instance engaging in OCB might also involve behaviors like working at the weekend which in turn might contribute to work-family conflicts (Halbesleben et al., 2009) or inferior in-role performance (e.g., Bergeron, 2007). Yet, OCB behaviors toward the benefits of co-workers, the specific focus of our study, might stem from rather-self-serving motives like impression management (Snell and Wong, 2007). These potential drawbacks of OCB have to be considered when interpreting our results.

### Practical Implications

We deem that the use of SOC behaviors in the work environment holds benefits for employees, with particular respect to their social functioning in team work environments. Previously, applied trainings that facilitate active development and implementation of SOC behaviors among employees have been introduced and evaluated (Müller et al., 2016; Becker et al., 2017). The overall intention of these SOC-based interventions is to enable employees to apply self-directed behaviors in the workplace, to maintain work ability, and to promote functioning on the job despite age-related changes. In SOC-based trainings, employees choose a specific goal to successfully cope with a critical job demand (selection), identify actions to achieve this goal in an optimal way (optimization), and consider alternative strategies in cases of external or internal hindrances during goal accomplishment (compensation). Our study results complement this line of thought in several ways: First, our findings inform practitioners that seek to advance SOC-based or similar interventions in occupational settings. Our findings corroborate, that SOC-based trainings should address social and organizational implications of self-directed behaviors at the work place. The development of self-directed behaviors should not be exclusively focused on individual needs but also be sensitive and responsive to the needs of co-workers. Consequently, the idiosyncratic development of SOC strategies should be complemented by reflections that expand the perspective of the trainees to the social and organizational consequences of their self-directed behaviors, e.g., through moderated discussions with co-workers or team supervision. Second, our study dispels potential concerns of managers against training approaches that focus on the promotion of individualized action strategies at the work place. Our findings indicate that individual benefits of the SOC use are not necessarily at the costs of their co-workers. Therewith our study corroborates the further implementation of occupational health interventions that build upon life-span perspectives in organizational practice.

## Conclusion

Our study shows that employees who report high use of loss-based selection are perceived positively by their colleagues in terms of OCB toward the benefits of the team and co-workers. We additionally showed that among senior teachers, the use of compensation strategies was positively related to OCB, whereas in younger teachers compensation was negatively related to OCB. Therewith, our findings contribute to a better understanding of the age-differentiated social effects of successful aging strategies in terms of SOC at work.

## Ethics Statement

The study was carried out in accordance with the recommendations to safeguard good scientific practice of the German Research Association (DFG).

## Author Contributions

AM drafted the manuscript. He contributed to the analysis, and interpretation of the data. MW developed the design and contributed to the data acquisition. He revised the manuscript critically for important intellectual content. He also contributed to the analysis, and interpretation of the data.

## Conflict of Interest Statement

The authors declare that the research was conducted in the absence of any commercial or financial relationships that could be construed as a potential conflict of interest.
